# Identification of Novel Adjuvants for Ebola Virus-Like Particle Vaccine

**DOI:** 10.3390/vaccines8020215

**Published:** 2020-05-10

**Authors:** Huapeng Feng, Sumiho Nakatsu, Tiago Jose da Silva Lopes, Masaki Imai, Seiya Yamayoshi, Makoto Yamashita, Tokiko Watanabe, Yoshihiro Kawaoka

**Affiliations:** 1 Division of Virology, Department of Microbiology and Immunology, Institute of Medical Science, University of Tokyo, Tokyo 108-8639, Japan; hpfeng@ims.u-tokyo.ac.jp (H.F.); sumiho.nakatsu@gmail.com (S.N.); mimai@ims.u-tokyo.ac.jp (M.I.); yamayo@ims.u-tokyo.ac.jp (S.Y.); yamakoto@ims.u-tokyo.ac.jp (M.Y.); 2 Department of Pathobiological Sciences, School of Veterinary Medicine, University of Wisconsin-Madison, Madison, WI 53711, USA; tiagojose.dasilvalopes@wisc.edu; 3 Department of Molecular Virology, Research Institute for Microbial Diseases, Osaka University, Osaka 565-0871, Japan; 4 Department of Special Pathogens, International Research Center for Infectious Diseases, Institute of Medical Science, University of Tokyo, Tokyo 108-8639, Japan

**Keywords:** Ebola vaccine, virus-like particle, adjuvants

## Abstract

Ebola virus disease is a severe disease, often fatal, with a mortality rate of up to 90%. Presently, effective treatment and safe prevention options for Ebola virus disease are not available. Therefore, there is an urgent need to develop control measures to prevent or limit future Ebola virus outbreaks. Ebola virus protein-based virus-like particle (VLP) and inactivated whole virion vaccines have demonstrated efficacy in animal models, and the addition of appropriate adjuvants may provide additional benefits to these vaccines, including enhanced immune responses. In this study, we screened 24 compounds from injectable excipients approved for human use in Japan and identified six compounds that significantly enhanced the humoral response to Ebola VLP vaccine in a murine model. Our novel adjuvant candidates for Ebola VLP vaccine have already been demonstrated to be safe when administered intramuscularly or subcutaneously, and therefore, they are closer to clinical trials than adjuvants whose safety profiles are unknown.

## 1. Introduction

Ebola virus disease (EVD), discovered in 1976 in South Sudan and Zaire (now known as the Democratic Republic of the Congo, DRC), is an acute disease with a fatality rate that varies from 25%–90% [1]. EVD outbreaks have only occurred in Africa until now, with the largest outbreak occurring in West Africa from 2014 to 2016, primarily affecting Guinea, Sierra Leone, and Liberia, and resulting in 28,646 cases and 11,323 deaths as of 30 March 2016 [2]. Currently, an Ebola outbreak has been occurring in the DRC since August 2018, with 3409 cases and 2236 death as of 22 January 2020 [3]. The genus Ebolavirus currently includes six species: Zaire ebolavirus (EBOV), Sudan ebolavirus, Reston ebolavirus, Taï Forest ebolavirus, Bundibugyo ebolavirus, and the newly identified Bombali ebolavirus [4,5,6]. EBOV was responsible for the 2014–2016 outbreak in West Africa and is responsible for the current outbreak in the DRC [7,8].

To prevent EVD, several types of Ebola vaccines are under development, including inactivated vaccines, viral vector vaccines, subunit vaccines, and DNA vaccines [9,10]. Among them, rVSV-ZEBOV, a replication-competent recombinant vesicular stomatitis virus (VSV)-based vector expressing the EBOV glycoprotein (GP) [11], was recently approved for medical use in the United States by the U.S. Food and Drug Administration (FDA) [12]. rVSV-ZEBOV was been shown to be highly effective in protecting against Ebola virus infection in Guinea when given as part of a Phase III trial during the 2014–2016 Ebola outbreak [13]. However, safety concerns remain [14,15]. In addition, three types of Ebola vaccines that are adenovirus-based vectors expressing Ebola GP are currently in human trials [16,17,18].

Ebola virus protein-based virus-like particle (VLP) vaccines consist of Ebola GP and the matrix protein VP40, which are produced in insect or mammalian cells [19,20,21,22]. Ebola VLP vaccines have demonstrated efficacy in animal models [19,21,23]. Baculovirus-derived subunit vaccines have been tested in several viruses [24,25,26,27,28]. The major advantage of this system is that it can yield high-levels of VLPs and be easily scaled-up for manufacturing [24,26]; however, protein-based vaccines may not be as immunogenic as live vaccines [29,30] and, therefore, the addition of appropriate adjuvants may improve their efficacy and enhance immune responses. Previously, we showed the usability of certain injectable excipients, approved for human use and with good safety profiles, to enhance the humoral immune responses to a seasonal influenza vaccine [31]. To explore adjuvant candidates that are safe and enhance the immunogenicity of Ebola VLP vaccine, here we screened 24 injectable excipients for adjuvant effects with an Ebola VLP vaccine in mice. We identified six compounds that enhanced Ebola virus-specific antibody responses in mice. Our findings will facilitate the development of effective adjuvanted Ebola VLP and inactivated whole virion vaccines.

## 2. Materials and Methods

### 2.1. Cells, Antibodies, and Gene Resources

Spodoptera frugiperda Sf9 cells (ATCC, CRL-1711) were cultured in Grace’s insect medium (Gibco) supplemented with 10% FBS, 1% penicillin-streptomycin, and 0.1% Pluronic F68 solution (Gibco) at 28 °C without CO_2_. High Five Cells (BTI-TN-5B1-4, Invitrogen) were maintained in Express Five SFM medium (Gibco) supplemented with l-Glutamine and 1% penicillin-streptomycin. We used Grace’s insect medium (Gibco) supplemented with 3% FBS, 1% penicillin-streptomycin, and 0.1% Pluronic F68 solution to amplify the recombinant baculoviruses.

Rabbit antisera against GP (C2023) and VP40, which have been described previously [32,33], and mouse monoclonal antibodies against Ebola GP protein (mAb 42/3.71 or mAb 12/1.1; kindly provided by Dr. Ayato Takada, Hokkaido University, Hokkaido, Japan) were used for Western blot analysis. A monoclonal antibody against Ebola GP protein (mAb 42/3.71) was also used as an ELISA positive control.

The GP and VP40 cDNAs sequences were consistent with those of Zaire ebolavirus Ebola virus/H.sapiens-wt/SLE/2014/Makona-G3750.2, which was isolated from a patient during the 2014–2016 Ebola outbreak in West Africa (GenBank accession No. KM233059).

### 2.2. Generation of Ebola VLPs in an Insect Cell Expression System

We generated the Ebola VLPs in a Bac-to-Bac baculovirus expression system (Invitrogen, Carlsbad, CA, USA) similar to that described elsewhere [20,34,35,36]. Briefly, the GP and VP40 genes were cloned into a transfer vector (pFastBac), designated as pFastBac-GP and pFastBac-VP40, respectively, digested with BamH I and Not I, and then the recombinant pFastBac-GP and pFastBac-VP40 were transformed into DH10Bac bacteria to generate the recombinant bacmids. The purified recombinant bacmids carrying the Ebola GP or VP40 gene were transfected into Sf9 cells with Cellfectin II reagent (Invitrogen) according to the manufacturer’s protocol. The recombinant baculoviruses (rBV), designated as rBV-GP and rBV-VP40 viruses, were harvested from the media on day 6 post-transfection, and expanded in Sf9 cells to make the working virus stocks. High Five Cells were co-infected with rBV-GP and rBV-VP40 viruses and were incubated in magnetic culture vessels (250 mL media/1 L vessel) at 28 °C. The culture supernatants were collected at 60 h post-infection and clarified by centrifugation at 1400× *g* for 15 min at 4 °C. Supernatants were concentrated and purified by ultracentrifugation at 133,900× *g* for 1.5 h at 4 °C through a 25% sucrose cushion, and the VLP pellets were resuspended in PBS. The purified VLPs were aliquoted and stored at −80 °C until use. The concentration of total proteins was determined with a BCA Protein Assay Kit (Thermo Fisher Scientific, Waltham, MA, USA) following the manufacturer’s instructions.

### 2.3. Detection of Ebola VLP Secretion by Transmission Electron Microscopy (TEM)

High Five Cells were co-infected with rBV-GP and rBV-VP40 viruses at a multiplicity of infection (MOI) of 10 at 28 °C. After 24 h, the infected cells were collected and fixed chemically as previously described [37,38]. Ultrathin (50-nm-thick) sections were stained with 2% uranyl acetate and Reynold’s lead. The areas of interest were chosen randomly based on where budding VLPs and infected cells were observed. The images were then acquired with a Tecnai F20 transmission electron microscope (FEI Company, Eindhoven, The Netherlands) at 200 kV.

### 2.4. Generation of Soluble GP Mutant as an ELISA Coating Antigen

The GP mutant T42V/T230V GP1-632∆muc was generated by overlapping PCR using the GP cDNA as a template as described elsewhere [39]. Briefly, the GP mutant was generated by deletion of the mucin-like domain (residues 312–462) and transmembrane domain (residues 633–676) and mutation of two N-linked glycosylation sites (T42V and T230V). The GP mutant construct was cloned into the modified transfer vector pFastBac-Thrombin-His to add a 6 × His-tag at the C-terminal of the GP mutant for purification. The recombinant plasmid pFastBac-GP mutant was transformed into DH10Bac bacteria to generate the recombinant bacmid, and the purified recombinant bacmid carrying the GP mutant-His was introduced into the Sf9 cells by using Cellfectin II reagent. The recombinant baculovirus expressing GP mutant-His was collected on day 6 post-transfection and expanded in Sf9 cells to generate the working virus stock.

High Five Cells were infected with 20 mL of the working stock of the rBV-GP mutant-His for 1 h at 28 °C. Then, 230 mL of fresh Express Five SFM medium (Gibco) supplemented with l-Glutamine and 1% penicillin-streptomycin were added, and the cells were incubated at 28 °C with mild magnetic mixing for 60 h. The supernatant was harvested by centrifugation at 5000× *g* for 5 min at 4 °C. The GP mutant was purified from the clarified supernatant by using His trap excel (GE healthcare) with the AKTA protein purification system (GE Healthcare). After purification, the buffer of the purified GP mutant was exchanged for PBS by use of Amicon^®^ Ultra 15 mL Centrifugal Filters (30 kDa cut-off, Merck Millipore, Burlington, MA, USA). The concentration of protein was determined with a BCA Protein Assay Kit (Thermo Fisher Scientific).

### 2.5. Characterization of Ebola VLPs by Immuno-EM

Purified VLP solution was absorbed to collodion-coated nickel grids and pre-fixed with 4% paraformaldehyde in 0.1 M cacodylate buffer for 1 min at room temperature (RT). After being washed three times with PBS, the samples were blocked with Blocking one (Nacalai) at RT for 15 min. The samples were subsequently incubated with anti-Ebola GP antibody (C2023) at RT for 1 h. After being washed 6 times with PBS, the samples were incubated with goat anti-rabbit IgG conjugated with 10-nm gold particles (BBInternational). After being washed with PBS, the samples were fixed with 2.5% glutaraldehyde at RT for 1 min and negatively stained with 1% Uranyl Acetate. The samples were then treated with carbon deposition and the areas of interest were chosen randomly. The images were acquired with a Tecnai F20 TEM (FEI Company, Eindhoven, The Netherlands) operated at 200 kV.

### 2.6. Coomassie Blue Staining and Western Blotting

Samples were added to 2 × SDS sample buffer (Novex) and heated at 95 °C for 5 min. Then, they were run on 4%–20% Mini-PROTEAN^®^ TGX™ precast protein gels (Bio-Rad), 10 µL/well, at 200 V for 37 min, two gels in parallel. The proteins on one gel were transferred to Immobilon-FL PVDF Membrane (Millipore) by using Trans-Blot SD Cell (Bio-Rad). The membrane was blocked with Blocking one (Nacalai) at RT for 30 min. For the primary antibodies, we used the rabbit anti-Ebola GP antibody (C2023) and rabbit anti-Ebola VP40 antibody. The primary antibodies were incubated overnight at 4 °C, followed by incubation with the horseradish peroxidase (HRP)-conjugated secondary antibodies [i.e., HRP-conjugated sheep anti-mouse IgG (GE Healthcare, NA931) and HRP-conjugated donkey anti-rabbit IgG (GE Healthcare, NA934)]. Reactions were detected with Amersham ECL Prime Western Blotting Detection Reagent (GE Heathcare), and the images were acquired by using the ChemiDoc Touch imaging system (Bio-Rad). The other gel was put into Quick-CBB PLUS (Wako) staining for 30 min and then rinsed with distilled water for 60 min. The gel was then subjected to the ChemiDoc Touch imaging system to acquire the images.

### 2.7. Immunization of Mice with The Ebola VLP Vaccine

Five-week-old female BALB/c mice were purchased from Japan SLC Inc. After one week of adaptation, the mice were immunized with a 1 μg dose of purified Ebola VLPs with or without compounds (100 µg/dose) via intramuscular injection into the gastrocnemius muscle (four mice/group). For the screen, mice were immunized three times with PBS, the Ebola VLP vaccine only, or the Ebola VLP vaccine plus test compound [100 µg/dose; except for ethanol, which was used at 10% (*v*/*v*)] by intramuscular administration in a 100 µL volume with a two-week interval between the vaccinations. On day 14 after the boost-immunization and day 14 after the third immunization, 400 μL of blood was collected from each animal via the facial vein by using a goldenrod animal lancet (5 mm), and sera were isolated for measuring Ebola GP-specific antibody titers.

### 2.8. Measurement of Virus-Specific Antibody Titers

Virus-specific antibody titers in the mouse sera were determined by using a modified ELISA as previously described [31,40,41,42]. Briefly, 96-well ELISA plates (IWAKI) were coated with 5 µg/mL of purified GP mutant protein solution, which was produced as described in the Supplementary Information, overnight at 4 °C (50 µL/well). The plates were blocked with 200 µL of 20% Blocking one (Nacalai) in water at RT for 1 h. After being blocked, the plates were washed once with PBS containing 0.05% Tween-20 (PBS-T), and then 2-fold serially diluted serum samples were added to the plates, followed by a 1 h incubation at RT. Bound IgG was detected by using peroxidase-labeled goat anti-mouse IgG (gamma) antibody, F (ab’) 2 fragment (Kirkegaard & Perry Laboratory Inc.) or horseradish peroxidase-conjugated anti-mouse IgG1 or IgG2a antibodies (Southern Biotech). After the plates were washed four times with PBS-T, 100 µL of 2, 2′-azino-bis (3-ethylbenzothiazoline-6-sulphonic acid) diammonium salt substrate solution was added to each well to initiate the color reaction, and the OD was measured at a wavelength of 405 nm. Naive mouse serum was used as the negative control and a monoclonal antibody against Ebola GP protein (mAb 42/3.71) as the positive control. The antibody titer was defined as the reciprocal of the highest serum dilution that produced an OD405 > 0.1 after correcting for the negative serum control.

### 2.9. Statistics

For the analysis of the GP-specific total titers, we transformed the values to the log 2 scale to stabilize the variance. Next, we used a one-way ANOVA followed by Dunnett’s test to compare each compound against the reference group (either the vaccine alone, or the vaccine plus AddaVax). We used the R statistical package (www.r-project.org), and the Multcomp package [43]. For the comparison of the IgG1 and IgG2a titers elicited by the different compounds, we used two-tailed, unpaired Student t-tests for each compound separately. In all cases, we considered the differences to be significant if the p-values were less than 0.05.

### 2.10. Ethics

All experiments with mice were performed in the biosafety level 2 containment laboratory in the Institute of Medical Science, the University of Tokyo (Tokyo, Japan) in accordance with the Regulations for Animal Care of the University of Tokyo and the Guidelines for Proper Conduct of Animal Experiments by the Science Council of Japan, and were approved by the Animal Experiment Committee of the Institute of Medical Science, the University of Tokyo (Approval No. PA15-101).

## 3. Results

### 3.1. Generation and Characterization of Ebola Makona VLPs in a Baculovirus Expression System

**Figure 1:**
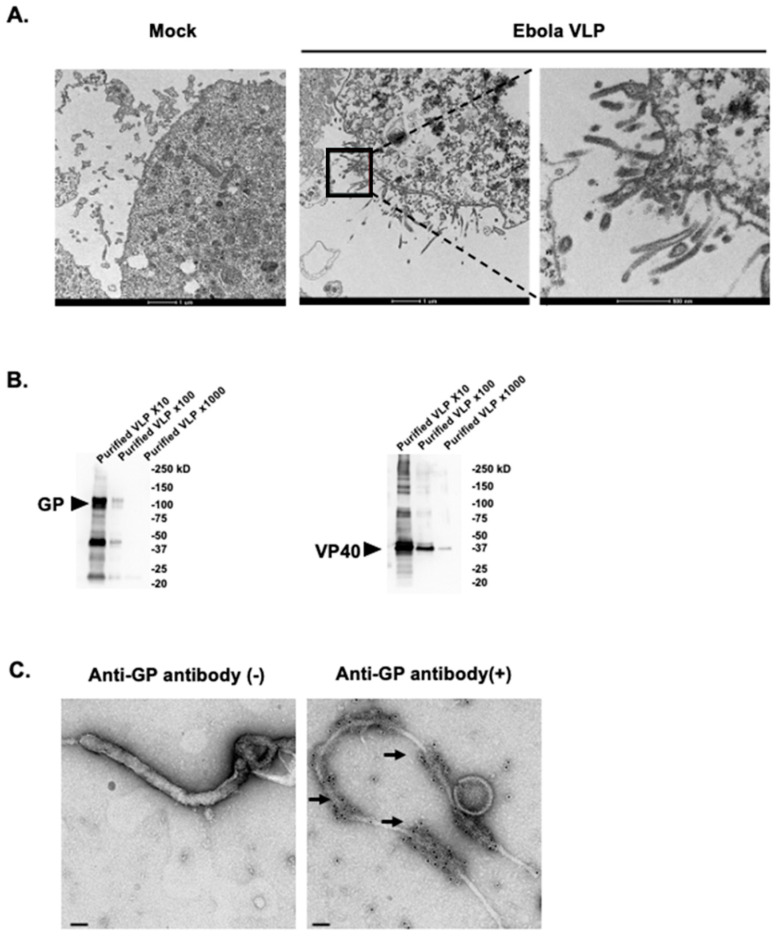
Generation and characterization of Ebola Makona virus-like particles (VLPs). (**A**) Budding of Ebola VLPs from insect cells. High Five Cells were infected with recombinant Baculovirus expressing GP protein (rBV-GP) and recombinant Baculovirus expressing VP40 protein (rBV-VP40). At 24 h post-infection, the cells were fixed with 2.5% glutaraldehyde. Ultrathin (50-nm-thick) sections were stained with 2% uranyl acetate and Reynold’s lead and were observed under a transmission electron microscope. (**B**) Western blot analysis of the purified Ebola VLPs subjected to SDS-PAGE followed by Western blot analysis with rabbit polyclonal antibodies against the Ebola VP40 and GP proteins. Purified VLP ×10, ×100, and ×1000 indicate the purified VLP diluted to 1:10, 1:100, and 1:1000. (**C**) Electronic microscopic analysis of the purified VLPs. The purified VLPs were immunogold stained for Ebola GP with a rabbit anti-GP polyclonal antibody as the primary antibody and a goat anti-rabbit IgG 10 nm gold as the secondary antibody, fixed, cut, and analyzed by means of electron microscopy. Arrows indicate 10 nm gold particles conjugated with goat anti-rabbit IgG. Scale bar indicates 50 nm.

We generated Ebola Makona VLPs composed of the Ebola GP and VP40 proteins in a Bac-to-Bac baculovirus expression system. The High Five Cells, which were co-infected with rBV-GP and rBV-VP40 viruses, produced filamentous particles from the cell surface as observed by TEM (Figure 1A). The filamentous VLPs were approximately 80 nm in diameter and 1000 nm in length, similar in size and morphology to virus particles observed in Ebola virus-infected cells [44,45]. The culture supernatant containing the Ebola Makona VLPs was concentrated by ultracentrifugation through a 25% sucrose cushion, and then the pellet was resuspended in PBS. The total protein concentration of the pellet was 5 µg/µL, as determined by using a BCA protein assay. The pellet was subjected to Western blot analysis, which showed that the Ebola Makona GP and VP40 proteins were present in the concentrated pellet (Figure 1B), indicating that the pellet contained substantial amounts of Ebola VLPs.

Since Ebola VP40 alone has been shown to form and be released as VLPs [20,21,37], we next examined whether the GP proteins were incorporated into the VLPs by using electron microscopy. Negative staining images showed that the concentrated VLPs contained filamentous particles (Figure 1C) that were identical in morphology to Ebola virus particles observed in Ebola virus-infected cells [44,45]. The immunogold staining of the VLPs with the anti-GP antibody showed that GP was present on the surface of the filamentous particles, whereas no signal was observed in the absence the anti-GP antibody (Figure 1C).

### 3.2. Identification of Six Compounds That Enhance the Humoral Response to the Ebola Makona VLP Vaccine in Mice

To identify novel adjuvants that enhance immune responses to Ebola Makona VLP vaccine, we screened 24 injectable excipients in a mouse model. These compounds have all been approved for intramuscular or subcutaneous administration in humans in Japan, including 14 compounds approved by the FDA (Table S1). Of the 24 compounds, 21 were found to have adjuvant effects on seasonal influenza vaccine in our previous study [31].

First, we performed an optimization experiment to determine the optimal dose of Ebola VLP vaccine for our screen by measuring levels of GP-specific antibodies in an ELISA with different doses of Ebola VLP vaccine. The soluble GP mutant was generated as described in the Supplementary Information (and Figure S1) and was used as an ELISA antigen. The commercially available adjuvant MF59-like AddaVax, a squalene-based oil-in-water nano-emulsion, was used as a positive control. We found that a dose of 1 µg of Ebola VLP vaccine alone elicited no or very low levels of GP-specific antibodies (Figure S2), whereas the GP-specific antibody response was significantly enhanced when the AddaVax adjuvant was administered to mice together with the Ebola VLP vaccine. In contrast, a dose of 3 µg of Ebola VLP vaccine induced high levels of GP-specific antibodies both in the presence and absence of AddaVax (Figure S2). Therefore, we decided to use the dose of 1 µg of Ebola VLP vaccine for our screen.

**Figure 2:**
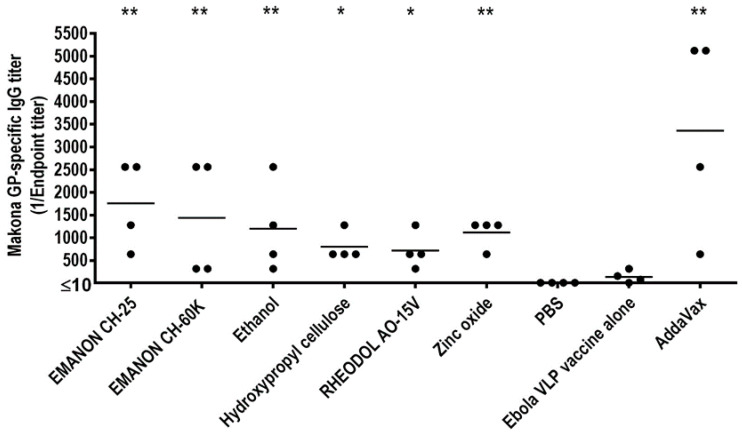
Screening adjuvant candidates for Ebola Makona VLP vaccine Three groups of six-week-old BALB/c mice (*n* = 4) were intramuscularly immunized with PBS, Ebola VLPs alone, or Ebola VLPs plus compound three times. Ebola VLPs plus AddaVax was used as a positive control. Blood was collected two weeks after the third immunization. Ebola GP-specific antibodies were assessed by using an enzyme-linked immunosorbent assay (ELISA) using purified His-tagged GP mutant as the coating antigen. Asterisks indicate that the antibody titer was significantly higher in mice immunized with the Ebola VLP vaccine plus the respective compound compared with the vaccine alone group (* *p* < 0.05; ** *p* < 0.01).

For the screen, mice were immunized three times with PBS, the Ebola VLP vaccine only, or the Ebola VLP vaccine plus test compound [100 µg/dose; except for ethanol, which was used at 10% (*v*/*v*)] by intramuscular administration in a 100 µL volume with a two-week interval between the vaccinations. Ebola VLP vaccine plus AddaVax was used as a positive control. On day 14 after the boost-immunization and day 14 after the third immunization, serum samples were collected from the immunized mice and subjected to ELISA to measure the titers of GP-specific antibodies. The antibody titers for the second immunization were relatively lower than those for the third immunization (Table S1). No antibody against Ebola GP protein was detected in the PBS group (Figure 2 and Table S1). Most of the mice immunized with the Ebola VLP vaccine alone produced no or low levels of GP-specific antibodies after three immunizations (Figure 2 and Table S1). For the statistical analysis of the GP-specific total titers, the values were transformed to the log 2 scale to stabilize the variance (Table S2). We defined hits as compounds that induced significantly higher antibody titers when combined with the Ebola VLP vaccine compared with the Ebola VLP vaccine alone group. According to these criteria, we identified six compounds that enhanced antibody production compared with Ebola VLP vaccine alone after three immunizations: EMANON CH-25 [polyoxyethylene (25) hydrogenated castor oil], EMANON CH-60K [polyoxyethylene (60) hydrogenated castor oil], ethanol, hydroxypropyl cellulose, RHEODOL AO-15V (sorbitan sesquioleate), and zinc oxide (Figure 2). These six hits are all novel adjuvant candidates for Ebola VLP vaccine. EMANON CH-25 induced the highest level of GP-specific antibodies after the third immunization (Figure 2 and Table S1).

### 3.3. Induction of Ebola Virus-Specific IgG1 and IgG2a Antibodies in Mice Immunized with the Adjuvanted Ebola VLP Vaccine

We next measured the Ebola virus-specific IgG1 and IgG2a titers in the serum samples collected from the immunized mice by using an ELISA because IgG1 and IgG2a are stimulated during Th2-type and Th1-type immune responses, respectively [46,47,48]. The IgG1 titers in the VLP vaccine plus zinc oxide and the VLP vaccine plus AddaVax groups were significantly higher than those in the VLP vaccine alone group (Table 1). Mice immunized with the VLP vaccine plus EMANON CH-25, EMANON CH-60K, ethanol, or zinc oxide raised significantly higher levels of IgG2a antibodies compared with the VLP vaccine alone group, whereas there was no significant difference between the IgG2a titers of mice immunized with the VLP vaccine plus AddaVax and those of mice immunized with the VLP vaccine alone (Table 1). We also calculated the IgG2a/IgG1 ratios. Those ratios for mice immunized with VLP vaccine plus AddaVax, EMANON CH-25, hydroxypropyl cellulose, or zinc oxide were 0.18, 0.49, 0.77, and 0.61, respectively (Table 1), suggesting that Th2-biased immune responses were induced in those groups. In contrast, the IgG2a/IgG1 ratio for the VLP vaccine plus ethanol group was 3.05, indicating the induction of Th1-biased immune responses in this group.

## 4. Discussion

Ebola VLP vaccine is a promising option for EVD prevention [19,21,23]. A baculovirus expression system has the advantages of easy use and easy scale-up for future manufacturing under Good Manufacturing Practice conditions [19]. Previous studies have demonstrated that Ebola VLPs activate dendritic cells, induce neutralizing antibodies, and confer effective protection against lethal challenge of Ebola virus in animal models [19,20,21,23,34,49,50]. High-dose Ebola VLP vaccines have been shown to provide complete protection from Ebola virus infection in mouse and nonhuman primate models [19,21,23]; however, the addition of appropriate adjuvants would reduce the vaccine doses and elicit enhanced immune responses [51,52]. Ideal adjuvants would be safe in humans and effectively enhance vaccine efficacy. Previously, we demonstrated that several injectable excipients, which are approved for human use in Japan, enhanced the efficacy of influenza vaccine against lethal virus challenge in a mouse model [31]. In the present study, we screened 24 injectable excipients, including 14 FDA-approved compounds (Table S1), and identified six novel adjuvant candidates that significantly enhanced the humoral response to Ebola VLP vaccine in mice (Figure 2), although the neutralizing antibody titers were not determined in this study. The safety of these compounds is expected to be high in humans given that they are already approved for human use. However, conjugation with Ebola VLP vaccine may affect the safety profile of the compounds, and therefore, it will be important to retest the safety of each compound in a future study. Nonetheless, they would theoretically move more quickly into clinical trials than compounds whose safety profiles have not yet been determined. These novel adjuvants are likely to be beneficial for inactivated Ebola whole virion vaccines [29].

IgG1 and IgG2a are stimulated during Th2-type and Th1-type immune responses, respectively [40,41,42]. Previous studies have shown that immunization of mice with Ebola VLPs strongly induces IgG2a antibodies but low levels of IgG1 antibodies [19,20]. Yet, a nonhuman primate study with a rabies virus-based bivalent vaccine showed that IgG1-biased humoral immune responses were beneficial for protection of the immunized animals against Ebola virus infection [53], and the authors specifically demonstrated that the protected animals had IgG2a/IgG1 ratios of less than 1.0, compared with the unprotected animals, whose IgG2a/IgG1 ratios were above 2.0 [53]. In the present study, we found that the addition of several adjuvant candidates, such as EMANON CH-25, hydroxypropyl cellulose, and zinc oxide, to the Ebola VLP vaccine induced both IgG1 and IgG2a antibodies in the immunized mice, and that the IgG2a/IgG1 ratios for these groups were below 1.0 (i.e., 0.49, 0.77, and 0.61, respectively) (Table 1). Taken together, our data show that the adjuvant candidates identified in this study not only enhance the production of Ebola GP-specific antibodies, but also induce the IgG subclass switch to IgG1, which would be beneficial for protection against Ebola virus infection.

## 5. Conclusions

Ebola VLP vaccines have experimentally exhibited efficacy in animal models; however, their immunogenicity is relatively low compared to that of live vaccines. To explore adjuvant candidates that are safe and enhance the immunogenicity of Ebola VLP vaccines, here we screened 24 compounds from injectable excipients approved for human use in Japan, and identified six compounds that enhanced Ebola virus-specific antibody responses in mice. Our findings are of value to the development of effective and safe adjuvanted Ebola VLP vaccines.

## Figures and Tables

**Table 1:** GP-specific IgG1 and IgG2a antibody titers of the six injectable compound hits in mice ^a^.

Immunogen	GP-Specific IgG1 Titers ^b^		GP-Specific IgG2a Titers		IgG2a/IgG1 Ratio
	Individual Titers	Mean Titers	Individual Titers	Mean Titers	
PBS	<10, <10, <10, <10	NA ^c^	<10, <10, <10, <10	NA	NA
Vaccine alone	40, 80, 40, <10	NA	40, 80, 20, <10	NA	NA
Vaccine + AddaVax	80, 320, 2560, 2560	1380.00 ^d^	40, 640, 160, 160	250.00	0.18
Vaccine + EMANON CH-25	2560, 640, 640, 80	980.00	640, 320, 640, 320	480.00 ^d^	0.49
Vaccine + EMANON CH-60K	320, 1280,20,80	425.00	1280, 160, 80, 160	420.00	0.99
Vaccine + Ethanol	320, 80, 40, 320	190.00	1280, 80, 640, 320	580.00 ^d^	3.05
Vaccine + Hydroxypropyl cellulose	80, 10, 1280, 80	362.50	160, 320, 320, 320	280.00 ^d^	0.77
Vaccine + RHEODOL AO-15V	40, 640, 80, 320	270.00	640, <10, 640, 160	NA	NA
Vaccine + Zinc oxide	320, 1280, 640, 640	720.00 ^d^	640, 320, 160, 640	440.00 ^d^	0.61

^a^ Six-week-old BALB/c mice were intramuscularly immunized with PBS, Ebola VLP Vaccine alone, Ebola VLP Vaccine plus compound, or Ebola VLP Vaccine plus AddaVax, three times with a two-week interval between immunizations. Four mice were used per group. The serum samples were collected two weeks after the third immunization to measure the GP-specific IgG antibody titers. ^b^ The GP-specific antibody titers were determined by use of an ELISA with purified GP mutant as the coating antigen. The optical density (OD) was measured at a wavelength of 405 nm. The antibody titer was defined as the reciprocal of the highest serum dilution that produced an OD405 > 0.1 after correcting for the negative serum control. ^c^ NA, not applicable since the antibody titers in one or more mice were <10. ^d^ *p* < 0.05, Vaccine + compound or Vaccine + AddaVax group versus Vaccine alone group.

## Supplementary Materials

The following are available online at https://www.mdpi.com/2076-393X/8/2/215/s1, Figure S1: Expression and characterization of the Ebola Makona GP mutant that was used as the coating antigen for the ELISA; Figure S2: Optimization of the vaccine doses used for the screen; Table S1: Resources and total IgG antibody titers of the injectable excipients used in this study (Excel file); Table S2: Antibody titers transformed to the log2-scale, which were used for the statistical analyses in this study (Excel file).
