# Supplementary material for: Identification of Novel Adjuvants for Ebola Virus-Like Particle Vaccine
Source: Vaccines (Basel). 2020 May 10;8(2):215. doi: 10.3390/vaccines8020215 (PMC7349346; doi:10.3390/vaccines8020215)
Supplement: Supplementary file 1 [file vaccines-08-00215-s001.zip › Feng_Ebola VLP vaccine_rev_Sup. Figs.pptx]

## Slide 1
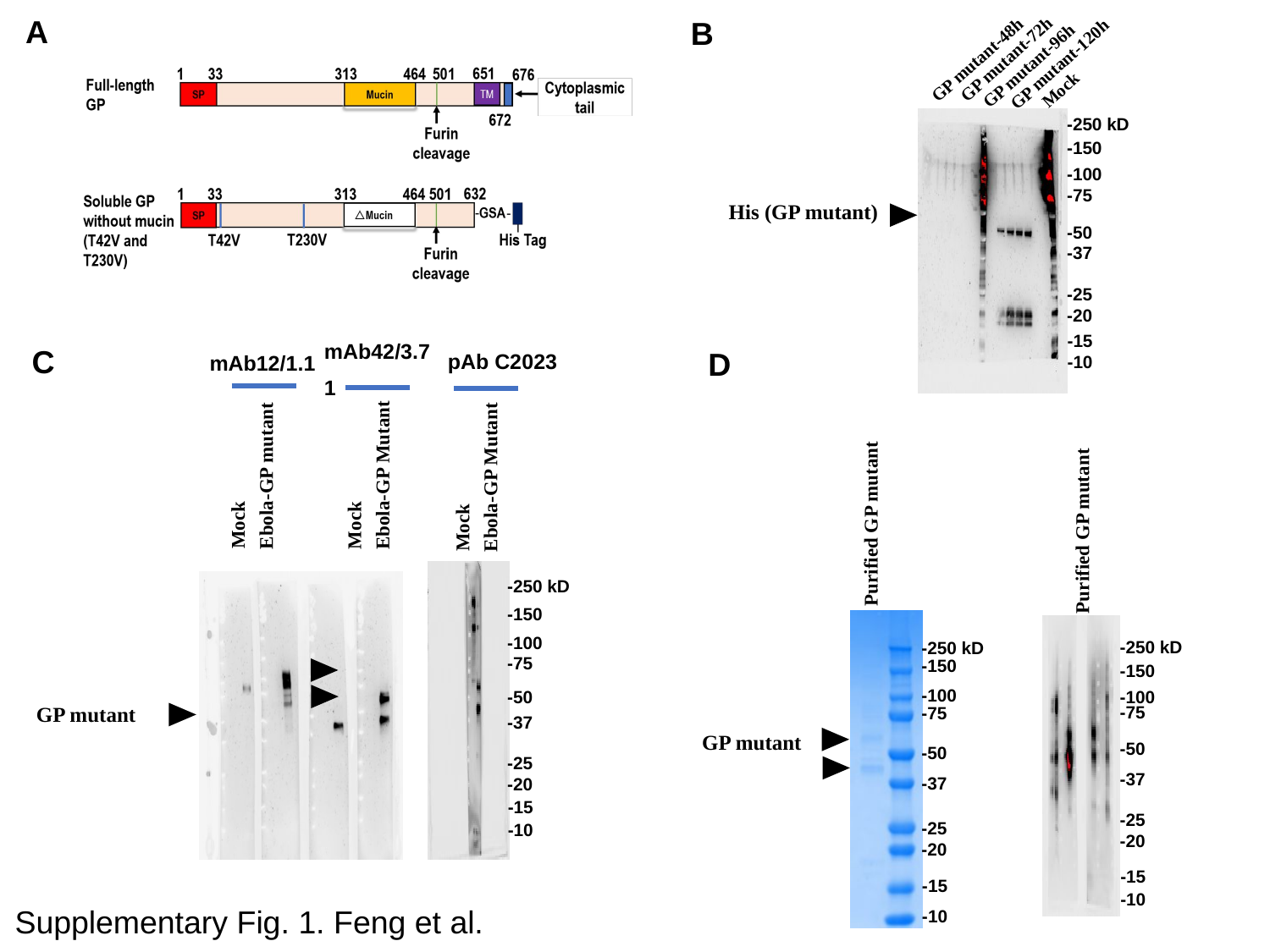

A
B
GP mutant-72h
GP mutant-48h
Mock
GP mutant-96h
GP mutant-120h
-250 kD
-150
-100
-75
His (GP mutant)
-50
-37
-25
-20
-15
pAb C2023
mAb42/3.71
mAb12/1.1
C
D
-10
Ebola-GP mutant
Ebola-GP Mutant
Ebola-GP Mutant
Mock
Mock
Mock
Purified GP mutant
Purified GP mutant
-250 kD
-150
-100
-250 kD
-250 kD
-75
-150
-150
-100
-50
-100
GP mutant
-75
-75
-37
GP mutant
-50
-50
-25
-37
-37
-20
-15
-25
-25
-10
-20
-20
-15
-15
-10
Supplementary Fig. 1. Feng et al.
-10

## Slide 2
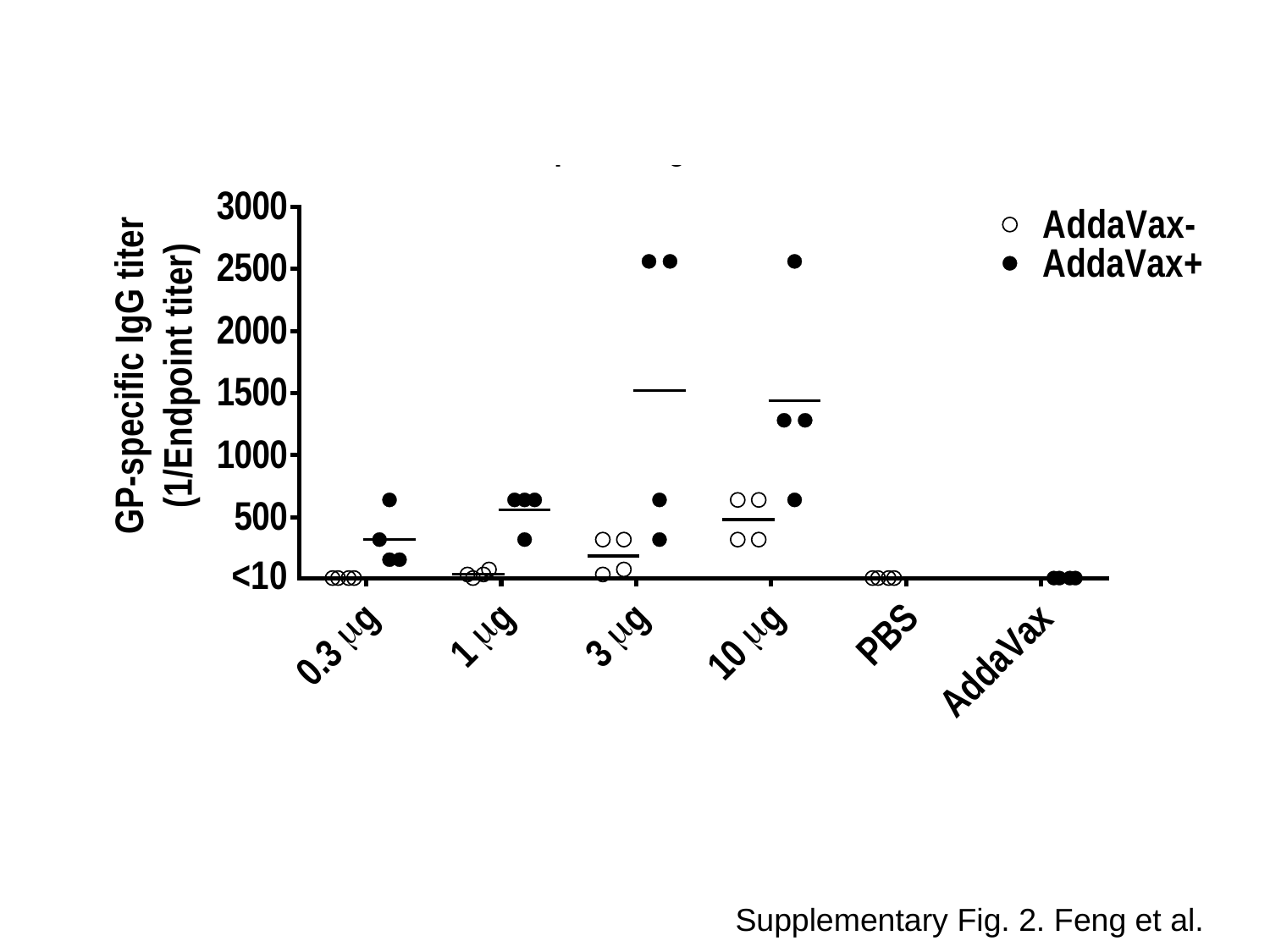

Supplementary Fig. 2. Feng et al.
