# Supplementary material for: Identification of Novel Adjuvants for Ebola Virus-Like Particle Vaccine
Source: Vaccines (Basel). 2020 May 10;8(2):215. doi: 10.3390/vaccines8020215 (PMC7349346; doi:10.3390/vaccines8020215)
Supplement: Supplementary file 1 [file vaccines-08-00215-s001.zip › Feng_Ebola VLP_rev_Sup info.docx]

**Supplementary Information**

- Supplementary Figure 1. Expression and characterization of the Ebola Makona GP mutant that was used as the coating antigen for the ELISA
- Supplementary Figure 2. Optimization of the vaccine doses used for the screen
- Supplementary Table S1. Resources and total IgG antibody titers of the injectable excipients used in this study (excel file)
- Supplementary Table S2. Antibody titers transformed to the log2-scale, which were used for the statistical analyses in this study (excel file)

**Supplementary text**

**Generation and characterization of the Ebola GP mutant**

For the coating antigen of an ELISA to measure the Mokona GP-specific antibody titer, we generated soluble Ebola GP protein by using the baculovirus expression system as described previously ([1-4](#_ENREF_1)) (Supplementary Fig. 1A). Western blot analysis with an anti-His antibody showed that the GP mutant protein was secreted into the culture supernatant of rBV-Ebola GP mutant-infected High Five Insect Cells (Supplementary Fig. 1B). The expression and proper conformation of the GP mutant was further confirmed with two monoclonal antibodies, mAb 12/1.1 and mAb 42/3.71, and a rabbit polyclonal antibody (C2023) against Ebola GP proteins by means of Western blotting. The mAb 42/3.71 and rabbit anti-GP serum reacted better with the GP mutant than did mAb 12/1.1, generating two bands of about 44 kDa and 60 kDa, which likely corresponded to the GP0 mutant and GP1 mutant, respectively (Supplementary Fig. 1C). The concentration of purified GP mutant was determined in a BCA protein assay and found to be 1.69 µg/µL. To further confirm the purity of the purified GP mutant, we performed Coomassie blue staining. As shown in Supplementary Fig. 1D, two bands (approx. 44 kDa and 60 kDa) representing the purified GP mutant were readily detected. These results demonstrate that soluble GP mutant protein fused with a His tag and expressed in insect cells can be easily purified and used as an ELISA antigen.

**Supplementary Figure legends**

**Supplementary Figure S1. Expression and characterization of the Ebola Makona GP mutant that was used as the coating antigen for the ELISA.**

(A) Schematic map of wild-type Ebola GP and the GP mutant. Briefly, the GP mutant was generated by deletion of the mucin-like domain (residues 312–462) and the transmembrane domain (residues 633–676) and by mutation of two N-linked glycosylation sites (T42V and T230V).

(B) Secretion of soluble GP protein. The High Five Cells were infected with rBV- GP mutant at an MOI of 5. The supernatants were collected at the indicated time and the GP mutant in the supernatant was analyzed by SDS-PAGE followed by western blotting with a mouse monoclonal anti-His tag antibody.

(C) Characterization of soluble GP protein with different anti-GP antibodies. The GP mutant in the supernatant was detected with three antibodies (mouse monoclonal antibodies mAb 42/3.71 or mAb 12/1.1 and a rabbit polyclonal anti-GP antibody C2023) by western blotting.

(D) Coomassie blue staining of purified Ebola GP mutant. A large amount Ebola GP mutant was produced by infecting the High Five Insect Cells with the rBV-GP mutant. The supernatant was collected at 60 h post-infection. The GP mutant in the medium was then purified by using His trap excel with the AKTA protein purification system and the elution buffer was exchanged to PBS by using Amicon® Ultra-15 Centrifugal Filter Units. The purified GP mutant was mixed with 2 × SDS sample buffer, heated at 70 °C for 10 min, and then subjected to SDS-PAGE followed by Coomassie blue staining and western blotting with rabbit polyclonal antibodies against Ebola GP protein as described in the Materials and Methods section.

**Supplementary Figure S2. Optimization of the vaccine doses used for the screen.**

Six-week-old mice were immunized twice with a two-week interval between immunizations. Four doses (0.3 µg, 1 µg, 3 µg, and 10 µg) of purified Ebola Makona VLPs with or without AddaVax were tested. Serum samples were collected from immunized mice two weeks after the second immunization and GP-specific antibody titers were measured by use of an ELISA. The black lines represent the mean antibody titers (n=4).

**References**

1. **Margine I, Palese P, Krammer F.** 2013. Expression of functional recombinant hemagglutinin and neuraminidase proteins from the novel H7N9 influenza virus using the baculovirus expression system. Journal of visualized experiments : JoVE**:**e51112.

2. **Warfield KL, Posten NA, Swenson DL, Olinger GG, Esposito D, Gillette WK, Hopkins RF, Costantino J, Panchal RG, Hartley JL, Aman MJ, Bavari S.** 2007. Filovirus-like particles produced in insect cells: immunogenicity and protection in rodents. The Journal of infectious diseases **196 Suppl 2:**S421-429.

3. **Ye L, Lin J, Sun Y, Bennouna S, Lo M, Wu Q, Bu Z, Pulendran B, Compans RW, Yang C.** 2006. Ebola virus-like particles produced in insect cells exhibit dendritic cell stimulating activity and induce neutralizing antibodies. Virology **351:**260-270.

4. **Warfield KL, Dye JM, Wells JB, Unfer RC, Holtsberg FW, Shulenin S, Vu H, Swenson DL, Bavari S, Aman MJ.** 2015. Homologous and heterologous protection of nonhuman primates by Ebola and Sudan virus-like particles. PloS one **10:**e0118881.
